# The histopathology and phenotypic variability in H syndrome

**DOI:** 10.1002/ccr3.1329

**Published:** 2018-01-25

**Authors:** David Dias‐Polak, Margarita Indelman, Reuven Bergman, Emily Avitan‐Hersh

**Affiliations:** ^1^ Department of Dermatology Rambam Health Care Campus HaAliya HaShniya St 8, Haifa POB 9602 Haifa 31096 Israel; ^2^ Laboratory of Molecular Dermatology The Bruce Rappaport Faculty of Medicine, Technion Rambam Health Care Campus HaAliya HaShniya St 8, Haifa POB 9602 Haifa 31096 Israel

**Keywords:** H syndrome, histopathology, phenotypic variability, recurrent mutation

## Abstract

Skin biopsy may be helpful in the diagnosis of H syndrome. A triad of dermal fibrosis, lymphocytic aggregates, and numerous CD68+, CD163+, S100‐positive, and CD1a‐negative dermal histiocytes is characteristic.

## Introduction

H syndrome is a recently described autosomal recessive disorder characterized by hyperpigmented, hypertrichotic, and indurated cutaneous plaques, predominantly located on the abdomen and lower extremities. Associated systemic manifestations include hepatosplenomegaly, congenital cardiac anomalies, hearing loss, hypogonadism, short stature, and hyperglycemia. H syndrome is caused by mutations in the gene SLC29A3 which encodes hENT3, a member of the human equilibrative nucleoside transporter family 1, 2, 3, 4, 5.

The histopathology shows dermal and subcutaneous fibrosis with numerous histiocytes, few plasma cells, and scattered and/or nodular aggregates of lymphocytes. The histiocytes display immunophenotypic similarity to Rosai–Dorfman disease (RDD), that is, CD68+, CD163+, S100+, and negative CD1a 4. Colmenero et al. 5 recently detected emperipolesis in addition to the RDD‐like phenotype. These unique histopathologic findings can assist in the diagnosis of H syndrome especially in view of its diverse clinical manifestations and clinical resemblance to morphea.

We describe herein a case with cutaneous findings suggestive of H syndrome in which the skin biopsy led to the correct diagnosis and consequently to the demonstration of a recurrent mutation with phenotypic variability.

## Case

A 15‐year‐old girl, daughter of consanguineous parents, was referred to our clinic due to asymptomatic cutaneous pigmentary lesions on her inner thighs for the past 4 years. She was diagnosed with hearing loss at the age of 3, attributed to recurrent otitis media. No similar findings were reported in her family.

**Figure 1:**
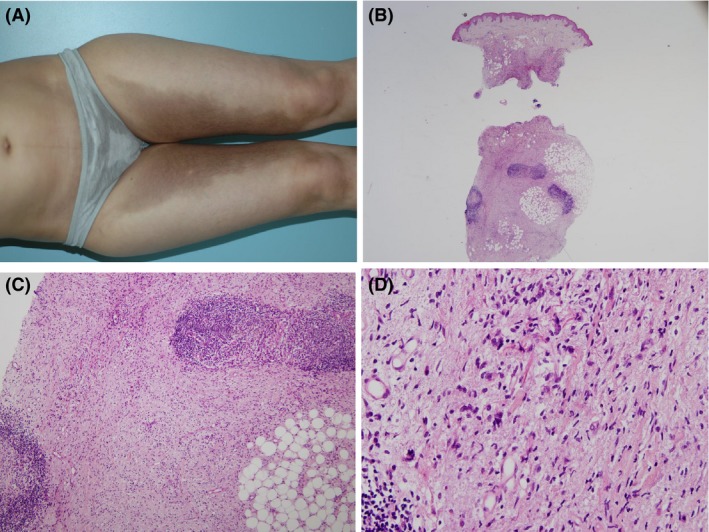
(A) Indurated hyperpigmented plaques distributed symmetrically on the inner thighs. (B) Histopathology of hyperpigmented plaque shows in the lower dermis fibrosis extending to the subcutaneous fat with several lymphoid nodules [hematoxylin and eosin (H&E) × 40]. (C) Higher magnification showing cellular fibrosis in the subcutaneous fat and lymphoid nodules (H&E × 100). (D) The cellular component of the fibrotic nodules is composed of spindle cells and histiocyte‐like cells with irregular nuclei (H&E × 400).

Dermatologic examination revealed well‐circumscribed, hyperpigmented, and hypertrichotic indurated plaques on the medial aspects of her thighs (Fig. 1). The remaining physical examination was unremarkable.

**Figure 2:**
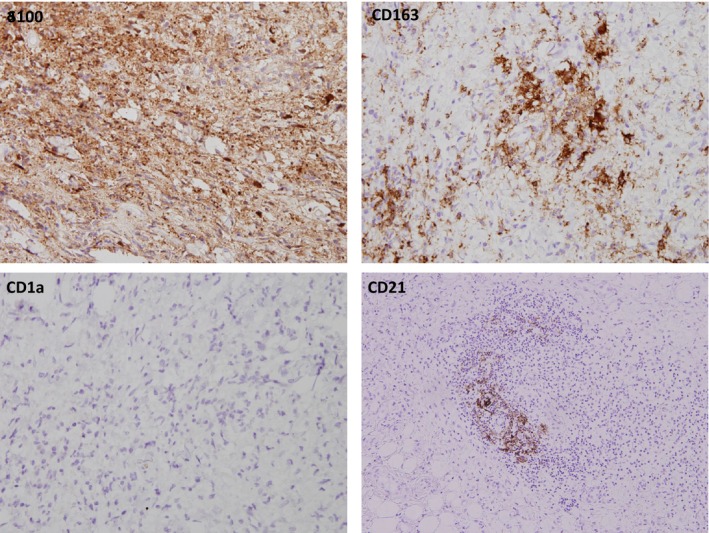
Immunohistochemical stains. Most of the cellular component stains for S100 protein and the histiocyte‐like cells stain for CD163. Immunostaining for CD1a is negative. The CD21 stain demonstrates aggregates of follicular center, dendritic cells which support the structure of a lymphoid follicle (immunoperoxidase, S‐100, CD163, CD1a × 400; CD21 × 200).

A 4‐mm punch biopsy was obtained from the involved medial thigh. The histopathologic examination demonstrated diffuse fibrosis in the lower dermis and subcutaneous fat (Fig. 1). In addition to many fibroblasts, there were numerous histiocyte‐like cells partly with irregularly shaped nuclei, nodular aggregates of lymphocytes, and scattered plasma cells (Fig. 1). The histiocytes stained positive for CD68, CD163, and S100 protein and negative for CD1a (Fig. 2). The lymphocytic aggregates harbored an equal number of CD20+ and CD3+ lymphocytes, but the Ki67 stain showed increased focal proliferative activity characteristic of germinal centers. The presence of germinal centers was also supported by the presence of aggregates of CD21+ follicular center dendritic cells (Fig. 2). Emperipolesis was not detected.

The cutaneous findings and histopathology were consistent with H syndrome 4, 5. Molecular studies performed on peripheral blood revealed a recurrent mutation c.G1309>A, which is expected to lead to glycine‐to‐arginine substitution in hENT3 (p.Gly437Arg) 1, 3, 6. Clinical and laboratory workup demonstrated a bilateral sensorineural hearing loss.

## Discussion

This case demonstrates the additional value of performing a skin biopsy to the diagnosis of H syndrome by showing characteristic findings of dermal fibrosis, lymphocytic aggregates, and numerous CD68+, CD163+, S100‐positive, and CD1a‐negative dermal histiocytes. This may be used to differentiate H syndrome from morphea, scleroderma, and POEMS syndrome which may resemble clinically. Phenotypic variability in H syndrome further emphasizes the need for diagnostic measures. The c.G1309>A mutation has been associated with multiple abnormalities including short stature, exophthalmos, dilated lateral scleral vasculature, hearing loss, congenital cardiac anomalies, hepatomegaly, hypogonadism, varicose veins, fixed flexion, hallux valgus, flat foot, malabsorption, and hyperglycemia 1, 6, 7, 8, 9, 10, 11, 12. Our patient had only hearing loss, a prominent feature of this syndrome 8. The paucity of other associated findings and the phenotypic variability increases the importance of skin biopsy in H syndrome.

## Authorship

Dr. David Dias‐Polak: contributed to the clinical analysis of the case. Margarita Indelman: contributed to laboratory workup of the case. Prof. Reuven Bergman: associated with the clinical analysis of the case. Dr. Avitan‐Hersh Emily: contributed to the clinical analysis of the case.

## Conflict of Interest

None declared.
